# Life cycle and morphogenesis of the hepatitis E virus

**DOI:** 10.1038/s41426-018-0198-7

**Published:** 2018-11-29

**Authors:** Kiyoshi Himmelsbach, Daniela Bender, Eberhard Hildt

**Affiliations:** 1 Department of Virology, Paul-Ehrlich-Institut, Paul-Ehrlich-Straße 51-59, 63225 Langen, Germany; 2 Deutsches Zentrum für Infektionsforschung (DZIF), Gießen-Marburg, Langen, Germany

## Abstract

Hepatitis E virus (HEV) is transmitted primarily via contaminated water and food by the fecal oral route and causes epidemics in developing countries. In industrialized countries, zoonotic transmission of HEV is prevalent. In addition, HEV is the major cause of acute hepatitis in healthy adults and can cause chronic hepatitis in immunocompromised patients, with pregnant HEV-infected women having increased mortality rates of approximately 25%. HEV was once an understudied and neglected virus. However, in recent years, the safety of blood products with respect to HEV has increasingly been considered to be a public health problem. The establishment of HEV infection models has enabled significant progress to be made in understanding its life cycle. HEV infects cells via a receptor (complex) that has yet to be identified. The HEV replication cycle is initiated immediately after the (+) stranded RNA genome is released into the cell cytosol. Subsequently, infectious viral particles are released by the ESCRT complex as quasi-enveloped viruses (eHEVs) into the serum, whereas feces and urine contain only nonenveloped infectious viral progeny. The uncoating of the viral envelope takes place in the biliary tract, resulting in the generation of a nonenveloped virus that is more resistant to environmental stress and possesses a higher infectivity than that of eHEV. This review summarizes the current knowledge regarding the HEV life cycle, viral morphogenesis, established model systems and vaccine development.

## Introduction

Hepatitis E virus is a member of the Hepeviridae family with a (+)- stranded RNA genome exhibiting a strain-dependent size of 7.2–7.4 kb. This nonenveloped virus has a diameter of 27–34 nm and a genome consisting of three open reading frames (ORFs). The 5′ untranslated region (UTR) (27 nucleotides) is capped with a 7-methylguanine and is followed by ORF1, which encodes the nonstructural proteins (NS) necessary for replication. ORF2 encodes the core protein for the viral capsid, whereas ORF3 partially overlaps ORF1 and encodes a viroporin-like protein. The genome of this virus harbors a 3′ UTR (65 nucleotides) that ends with a poly(A) tail and mirrors the mRNA structure. HEV infection normally causes self-limiting acute hepatitis, with a fatal case rate of under 0.1% in healthy patients, and chronic infection in immunocompromised patients. For pregnant women, especially those who are infected during the third trimester, the mortality rate is approximately 25%. The reason for this increased mortality rate is still not fully understood, but one possible explanation may be increased estrogen levels during pregnancy^1^. HEV1 and HEV2 infection during pregnancy leads to an increased risk of miscarriage, preterm delivery, and still birth. In patients suffering from chronic liver disease (e.g., hepatitis B virus infections), these infections often progress to liver failure, with a mortality rate of 27%^2,3^. In addition to the hepatic manifestations, several extrahepatic manifestations have been reported, including neurological (e.g., Guillain-Barré syndrome and encephalitis) and renal manifestations (e.g., IgA nephropathy). The virus is transmitted via the fecal oral route and was previously thought to only be a problem in developing countries. In these mostly epidemic areas, HEV transmission occurs from human to human through contaminated water due to poor hygiene. However, increased testing in different countries has revealed that HEV is also widespread in Southeast Asia, Africa, USA and in Europe. In South, Central and Southeast Asia, the Middle East, Africa and Mexico HEV genotypes 1 and 2, which are restricted to humans (HEV1 and HEV2) are the most prevalent genotypes. In these areas, contamination of water supplies with human feces is the cause of the spread of this virus. In contrast to HEV1, which that is most widespread, HEV2 is only found in Africa (apart from one outbreak in Mexico at the end of the last century). The most prominent genotype in Europe (genotype 3) displays a seroprevalence of between 5% and 20%, while in the US the seroprevalence is estimated to be approximately 9%. However, seroprevalence studies vary significantly with respect to the assay used and the geographical region being studied^4–6^. HEV3 and HEV4 are zoonotic viruses. In industrialized countries, HEV3 is endemic and is frequently transmitted via the consumption of undercooked pork as a foodborne zoonosis or originates from autochthonous cases. Additionally, infections can occur from eating meat from wild animals, such as deer, wild boar or rabbits. In the last decades, the situation in China has changed from a high-endemicity pattern characterized by frequent HEV1-associated outbreaks to a low-endemicity pattern with sporadic HEV4 infections. The epidemic outbreaks of HEV in other parts of Asia and Africa indicate the need for advanced preventive and acute treatment methods to counteract HEV infections.

Currently, the Hepeviridae family is subdivided into *Orthohepevirus* A–D. *Orthohepevirus* A encompasses seven genotypes, with genotypes 1 and 2 only infecting humans, while genotypes 3–7 are zoonotic^7^. More distant from human HEV is the avian-specific Orthohepevirus B, Orthohepevirus C (infects mink and ferret), and Orthohepevirus D (infects bats). A variety of model systems for the study of HEV have been developed. Virus-like particles (VLPs) arising from the self-assembly of the core protein (ORF2) were one of the first models used to study the structure of the virus, its entry process, and its vaccine potential. The establishment of infectious cDNA clones and replicon systems for cell culturing and the development of persistently infected cell lines has greatly improved the knowledge regarding HEV, although these systems have some disadvantages, such as low viral titers. Furthermore, the lack of a robust animal model is a major drawback, although recent reports regarding the infection of mice, pigs, rats, chicken, and tree shrews by HEV will help to provide a more detailed understanding of the HEV infection process^8,9^. In this review, the current knowledge of the hepatitis E virus life cycle, the established model systems, and the status of vaccine development will be discussed.

## HEV entry

**Fig. 1:**
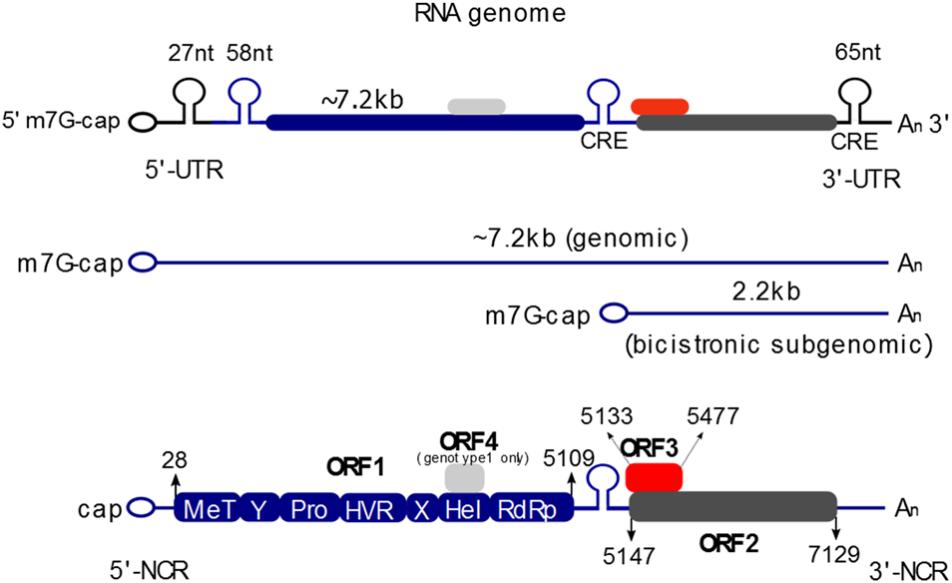
**Schematic illustration of nonenveloped HEV (found in the feces of infected patients) and of the quasi-enveloped form (found in the serum of infected patients and in cell culture supernatant of HEV-replicating cells)**

The hepatitis E virus can present as either a nonenveloped virus, where the capsid shell interacts with the surrounding environment, or as a quasi-enveloped virus, where the capsid is coated with an exosomal membrane (Fig. 1). Although both viral forms are infectious, the nonenveloped virus is 10 times more infectious than the quasi-enveloped form^10,11^. Negative contrasting and immunoelectron microscopy have shown that the nonenveloped virus has a diameter of ~30 nm, whereas the larger exosomal coated eHEV is ~40 nm in diameter^11,12^. The infectivity of HEVs derived from sera and cell culture supernatants was observed to be significantly lower than that of HEVs derived from stool and urine, reflecting the presence of quasi-enveloped and nonenveloped HEVs in the former and latter sample types, respectively^10,13^.

Viral nucleic acids are encased by a capsid and that is eventually covered by a membrane to protect the viral genome from environmental damage, which also facilitates viral binding and entry into host cells. Thus, the membrane must be stable enough to protect the viral nucleic acid but labile enough to undergo conformational changes upon contacting a host cell to promote its entry into the cell and allow for the release of the viral genome for replication. For enveloped viruses, the cellular uptake step is relatively well characterized, starting with the attachment of the virus to the cell surface, which normally results in the recruitment of several factors necessary for viral uptake by receptor mediated endocytosis.

For nonenveloped viruses, the entry process can be very different. Although the initial cellular uptake steps of nonenveloped viruses may be similar to those of enveloped viruses, such as attachment and the recruitment of several factors to the site of entry, no membrane fusion can occur. In these viruses, a conformational change of the core protein often occurs that results in the penetration of the outer membrane. The bottleneck for HEV is that a high multiplicity of infection is necessary to establish an ongoing infection. Unfortunately, the production of HEV in cell culture reaches poor titers of ~10^6^ genomes/ml.

The uptake mechanisms for most viruses are frequently based on endocytosis, including clathrin-mediated endocytosis (CME), caveolae-mediated endocytosis, and macropinocytosis. CME is characterized by the polymerization of clathrin at the cytoplasmic phase of the membrane inducing the formation of an invagination and a subsequent endosome. Because hepatitis E virus can exist as either a nonenveloped HEV or a quasi-enveloped eHEV, it was unclear whether both forms are internalized by the same entry mechanism. As described below, a number of studies have shown that the entry mechanisms for both forms appear to differ, with recent findings indicating that at least HEV uptake depends on CME^10,14^.

Due to a lack of robust infection systems, the initial investigations of HEV cell entry were performed by utilizing *E. coli*-derived HE-VLPs. He et al.^15^ used a truncated core protein p239 (aa 368–606), which was described to bind and enter HEV-susceptible cell lines (Huh7, HepG2, PLC/PRF5, and A549), preventing them from subsequent infection with HEV. Experiments using fluorescently labeled VLPs derived from full-length ORF2 fused to GFP suggested that HEV entry occurs via clathrin-mediated but not caveolin-dependent endocytosis, since only inhibitors for the clathrin-dependent endocytosis prevented viral entry^16,17^. Other studies using HE-VLPs showed the need of heparan sulfate proteoglycans in viral binding and uptake^16,17^. A recent report provided evidence for the involvement of the binding of ORF2 to the asialoglycoprotein receptor (ASGR) in the HEV entry process^18^. A systematic ORF2 interactome analysis based on split-ubiquitin, yeast-two-hybrid screening, and pull-down experiments confirmed the interaction of ORF2 with ASGR2 for HEV genotypes 1 and 4 (ref. ^19^). However, the direct interaction of  ORF2 with other entry factors described as being relevant has not been confirmed, including heat-shock cognate protein 70 (HSC70), glucose-regulated protein 78 (GRP78), or heat-shock protein 90 (HSP90)^20^.

The entry of eHEV and of the nonenveloped HEV form occurs with different kinetics. Quasi-enveloped HEV shows a slower entry kinetic compared to nonenveloped HEV. In one study, 90% of the maximal binding of nonenveloped particles was reached 3 h after infection, whereas eHEV required 6 h. Moreover, the binding efficiency of eHEV was ten-fold lower than that observed for naked HEV, resulting in a 2.7-fold lower infectivity of eHEV^10^. The entry mechanism was further investigated by the depletion of caveolin and clathrin using a siRNA approach and through the use of inhibitor molecules. These assays revealed that the infectivity of HEV and eHEV was not reduced when caveolin was blocked, whereas blocking clathrin had an inhibitory effect on the entry of both viral forms. In addition to the dependence on clathrin for cell entry, the necessity of endosomal acidification for viral entry was also demonstrated, excluding the direct penetration of the virus at the plasma membrane^10,15^. Furthermore, Yin et al.^10^ reported that depletion of Rab5 and Rab7 resulted in a 20% and 70% reduction in viral infectivity, suggesting that early (Rab5-positive) and late (Rab7-positive) endosomes have important functions in the establishment of viral infection. However, blocking of endosomal acidification by bafilomycin A (BFLA) and NH_4_Cl treatment could not block the entry of HE-VLPs^16^ corresponding to the nonenveloped form. This finding was corroborated for nonenveloped HEV by Yin et al.^10^ in 2016, whereas eHEV infection was found to be blocked by BFLA and NH_4_Cl. The pORF2-mediated interaction with heat-shock cognate protein 70 (HSC70), glucose-regulated protein 78 (GRP78), or heat-shock protein 90 (HSP90) has been shown to lead to budding into clathrin-coated pits that eventually leads to the uptake of nonenveloped particles^20,21^. Taken together, this highlights distinct entry mechanisms for HEV and eHEV, with eHEV entry utilizing the classic endosomal route.

## Replication

**Fig. 2:**
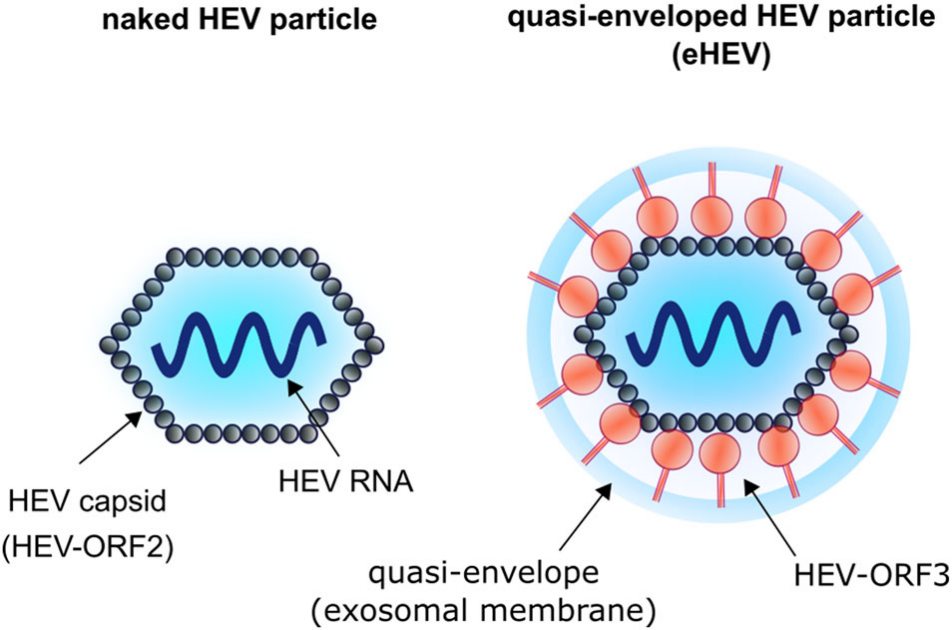
Genome organization of the hepatitis E virus. The 5′ end of the (+)-stranded RNA genome is capped with 7-methylguanosine (7 mG), while the 3′ end is polyadenylated (poly(A)). Open reading frame 1 (ORF1) encodes nonstructural proteins, including a methyltransferase (MT), papain-like cysteine protease (Pro), hypervariable region (HVR), helicase (Hel), and an RNA-dependent RNA polymerase (RdRp), as well as two regions of unknown function (Y-domain (Y), and X-/Macrodomain (X)). ORF2 encodes the core protein that forms the capsid, and the ORF3 protein is essential for viral release via the ESCRT pathway

The HEV genome resembles mRNA, with a 5′ cap and a 3′ poly-A tail structure (Fig. 2). A small noncoding region (NCR; 1-25 bp) is located at the 5′ end of the genome and is followed by open reading frame 1 (ORF1), encoding the nonstructural proteins necessary for replication (5109 bp) with a predicted molecular mass of ~185 kDa^22^. ORF1 and the two bicistronic overlapping reading frames ORF2 and ORF3 are separated by a small cis reactive element (CRE site). The 3′ end also contains an NCR and a poly (A) tail. After the polyprotein is translated, the replication of the viral RNA by RdRp proceeds with the synthesis of the negative strand RNA. Based on the (−)-strand, two different RNAs are synthesized, the full-length genomic RNA and a 2.2 kb subgenomic RNA^23^. The genomic RNA serves as a template for ORF1 translation and is packaged into viral particles or serves as a template for the synthesis of additional negative strand RNA, whereas the subgenomic RNA serves as a template for the translation of the capsid protein (72 kDa) and the ORF3 protein (13 kDa).

ORF1 is translated directly from the viral RNA, but it is not yet clear if it exerts its function as a multidomain polyprotein or if it must be cleaved into to obtain functional proteins. Based on sequence homologies, the polyprotein was proposed to consist of seven domains, starting with the methyltransferase followed by the Y-domain, PCP-domain, hypervariable-domain, pro-domain, X-domain, helicase-domain, and ending with the RNA-dependent RNA polymerase (RdRp). The expression of ORF1 in insect cells resulted in the translation of a large 110 kDa polyprotein that has guanine-7-methyltransferase and guanylyl transferase activities^24^. However, the baculovirus-driven expression of ORF1 in *T. ni* cells (*Trichoplusia ni*) by Shegal et al.^25^ resulted in the formation of an ~186 kDa polyprotein and the time-dependent appearance of cleavage products that were detectable by N- and C- terminal-specific antibodies^25^. The in vitro and in vivo expression of ORF1 using a vaccinia virus-based approach resulted in the synthesis of a 185 kDa protein, which formed ~107 and ~78 kDa cleavage-product proteins after a longer incubation time (24–36 h). Overexpression of the nonstructural polyprotein ORF1 in HepG2 cells also resulted in the expression of a 186 kDa protein that could be detected with methyltransferase-, helicase-, and RdRp-specific antibodies, but without the appearance of cleavage products. The expression of ORF1 in a prokaryotic system resulted in the production of an ~212 kDa GST-fusion protein, but no cleavage products could be observed. The ORF1 polyprotein has been observed to harbor conserved cleavage sites for thrombin and factor Xa, mutations in either of which impaired viral replication in cell culture, suggesting that factor Xa and thrombin are important for viral replication and processing of ORF1^26^.

The HEV helicase has NTPase activity and unwinds duplex RNAs in the 5′ to 3′ direction. Studies have shown that the NTPase removes gamma- and beta-, but not alpha-phosphate groups from the 5′ end of the RNA, with the removal of the gamma phosphate group being a prerequisite for the capping of cellular and viral RNAs^27^.

The HEV RdRp is essential for viral replication and shows different template affinities in vitro. The weakest binding affinity of RdRp was measured for the subgenomic promoter (SgP), followed by the 3´ end, and the highest binding was detected for the 5′ end. Surprisingly, cooperative binding was detected for the SgP and the 3′ end, which may be a mechanism to prevent binding to the 5′ end of the (−)-strand, which also exerts a strong RdRp affinity^28^. Homodimerization of the RdRp has been described demonstrated for HEV by yeast-2-hybrid screening data, and this activity has also described for other viruses^29^. Because RdRp lacks proofreading activity, the virus can evolve fast and can easily escape treatments with nucleoside analogs^30^. Because HEV rdRp is a highly error-prone RNA polymerase, it is astonishing that a target sequence for miR-122 is conserved in HEV1 genomes. miR-122 facilitates HEV replication, and inhibition or depletion of miR-122 leads to a drastic reduction in HEV replication. It is believed that miR-122 binds to the target site in the HEV genome to facilitate HEV replication^31^. As the miR-122 inhibitor miravursen is currently being tested in clinical trials for HCV, it is tempting to speculate whether this substance could be used to impair HEV replication. A very simple approach to affect HEV replication is based on the application of zinc salts, which impair HEV replication by the direct inhibition of the RdRp and could be used to support more specific antiviral therapies^32^.

The junction region between ORF1 and ORF2 contains a highly conserved stem loop structure and contains the promoter for a capped bicistronic subgenomic RNA encoding the ORF2 and ORF3 proteins. Mutations in the stem–loop region inhibit replication^33,34^. The SgP showed a stronger expression in vitro compared to the 3' NCR, which would result in an increase in core protein synthesis and a restricted synthesis of the (−)-strand. Kumar et al. reported a 2–3-fold excess of (+)-strand RNA versus the (−)-strand RNA, since the antigenomic strand displays an intermediate state. However, the existence and function of the prolonged RNA has not been investigated in HEV-replicating cell cultures or even infected individuals.

## Morphogenesis and release

ORF3 has been demonstrated to be crucial for the release hepatitis E virus from infected cells, but plays no role in the infection, replication, and assembly of the virus. The ORF3 protein is phosphorylated and interacts with the capsid protein and cellular components, such as the cytoskeleton, a1-microglobulin/bikunin precursor, tumor susceptibility gene 101 (TSG101), and src-homology domains^35–37^. ORF3 appears to fulfill a variety of different functions in the viral life cycle. Ding et al. described the ORF3 protein as an ion channel, with the C-terminus facing into the ER-lumen and the phosphorylated N-terminus reaching into the cytosol, similar to Vpu from HIV (human immunodeficiency virus) or M2 from IAV (influenza A virus). Measuring the ion current by voltage clamp experiments after alanine scanning mutagenesis resulted in the identification of the ORF3 positions [CCC11-13AAA] and [IFI59-61AAA], which are essential for ion flux, whereas mutations in the PXXP motifs did not influence ion channel capacity^38^.

**Fig. 3:**
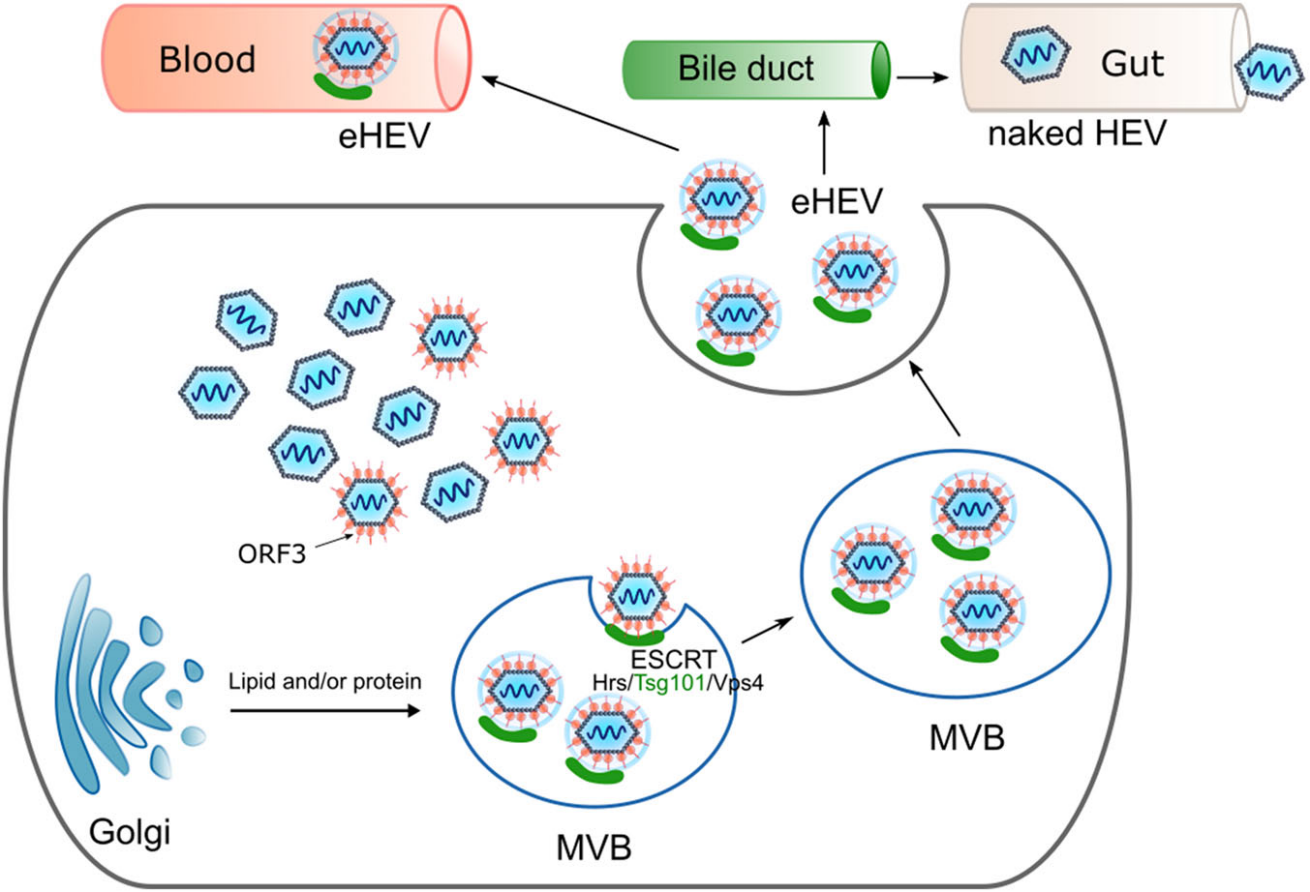
Schematic representation of the MVB-dependent release of the hepatitis E virus via exosomes. The PXXP motifs in the ORF3 protein interact with components of the cellular ESCRT machinery to facilitate the release of quasi-enveloped viruses into the bloodstream. After encountering bile, the viral envelope is removed and the nonenveloped virus is released via feces and urine

An ORF3 deletion mutant generated by mutating the third internal start codon was still able to replicate the genome but could no longer efficiently release virions. Furthermore, two PSAP motifs (late domain) in ORF3 were reported to be indispensable for viral release. The PXXP motif at amino acid position 95–98 is conserved among all HEV genotypes, including avian HEV. Investigations of avian HEV have also shown a predominant role of the PXXP motif (PREPSAPP) in the release of the virus in chicken cells and that the interaction of TSG101 with ORF3 depends on the proline residues in the PSAP motif but not the PREP motif^39^. Some strains, such as pJE03-1760F/wt, even contain two PXXP motifs. While in this case the mutation of one PXXP motif alone had no effect on the release of viral particles, the mutation of both motifs together resulted in a strong reduction of viral secretion. The double mutant no longer released viruses that possessed the expected density of 1.15 g/ml for quasi-enveloped particles but rather exhibited a density of 1.27 g/ml that corresponded to the nonenveloped virus (Fig. 3). This result suggests that disruption of this interaction prevents the release of enveloped viral particles, indicating that the ESCRT complex plays a pivotal role in the release of HEV^40–43^. The inhibition of the ORF3/TSG101 interaction by a cyclic peptide inhibitor impairs HEV release^44^. The interaction of the ORF3 protein with different cellular proteins was described to be mediated by their cellular SH3-domains^45,46^. The use of Bafilomycin resulted in an over a 200% increased release of virions, and in concordance with this result, a block in endosome formation by the ceramide biosynthesis inhibitor GW4869 reduced the release of virions to 74%. Moreover, TEM and immunoelectron microscopic analyses revealed that membrane-associated HEV was present in the MVBs^43^. Measuring the densities of viruses obtained from cell culture supernatants, sera, feces, and urine revealed that cell culture- and serum-derived viruses possesses a density of 1.10–1.15 g/ml, while viruses derived from urine and feces have a density of 1.20–1.27 g/ml. A detailed analysis of the quasi-enveloped particles revealed that they had a diameter of 39.6 ± 1.0 nm. After treatment with detergent and protease, the diameter of these VLPs shifted to 26.9 ± 0.9 nm. The size of these particles indicates that the capsids of HEV particles are individually wrapped by lipid membranes corresponding to those of exosomes^47^.

While the ORF3 protein is detectable on serum-derived viruses, it is absent on feces derived ones.

The treatment of serum- or cell culture-derived eHEV with detergent and protease can destroy the exosomal shell, making the virus accessible to neutralization by ORF2- and ORF3-specific antibodies. By using an ORF3-specific monoclonal antibody targeting the C-terminus, it was possible to pull down HEV from cell culture medium and serum, indicating that ORF3 is part of the virion. In contrast, it was not possible to precipitate the virus with the ORF3 antibody from feces^13^, indicating that no viral proteins were present on the surfaces of the enveloped virions. However, removal of the envelope by detergent treatment increases the infectivity of these viruses^10^. Thus, covering the virus in an exosomal shell is a strategy used to protect the virus from the immune system in the blood stream.

## Classification of the hepatitis E viruses

The most accepted classification of hepatitis E viruses has involved their categorization into four major genotypes, with genotype 1 (Pakistan Sar55 strain—M80581) and genotype 2 (Mexican strain—M74506) only infecting humans, while genotype 3 (US strains—AF082843) and genotype 4 (Chinese T1 strain—AJ272108) are zoonotic^22,47–49^. Genotypes 1 and 2 are relatively conserved because they only infect humans, but genotypes 3 and 4 are much more diverse since they have a broader host spectrum^50^. The identification of more HEV isolates from different species, such as trout, deer, rabbit, chicken, mongoose, rats, bats, ferrets, and camels has demonstrated the need for a new taxonomic system of the Hepeviridae family^51–53^. Unfortunately, for newly detected viral strains, there are frequently no full-length sequences available. Thus, Schlauder et al. used a region at the 5′ end in ORF1 for phylogenetic analysis, resulting in the classification of nine groups. The most recent division by the International Committee on Taxonomy of Viruses (ICTV) followed the suggested structuring by Smith et al. in 2014, which divided the family Hepeviridae into the genera Orthohepevirus and the more divergent Piscihepevirus. Orthohepeviuses are further subdivided into Orthohepevirus A–D, with the Orthohepevirus A harboring the genotypes (1–7) that infect humans. Avian HEV was first described in chickens in the US and was classified in the group Orthohepevirus B^51^. The group Orthohepevirus C contains HEV that infect ferrets and minks, while Orthohepevirus D includes HEV isolated from bats^7,54^.

## Models

### Cell culture systems

Suitable and efficient cell culture systems are extremely helpful in viral research for investigating various aspects of the viral life cycle. Various attempts have been undertaken to propagate HEV in multiple cell types. In 1995, Huang et al. reported for the first time the passaging of the 87 A strain isolated from 2BS cells from the feces of an HEV-infected patient in A549 lung carcinoma cells. The virus was detected via PCR alone, and no viral protein was assessed^47^. In addition, the cultivation of primary liver cells isolated from experimentally infected macaques attempted but could not amplify HEV in cell culture^55^.

Another approach that is well established for other single-stranded RNA viruses (e.g., hepatitis C virus) is the use cloned viral genomes that were transcribed and capped in vitro. This viral RNA can be used to transfect various cell lines. The successful transfection of HepG2 cells with in vitro transcribed RNA from a full-length cDNA clone was first described in the year 2000, and it displayed a transient cell culture model. Indeed, rhesus monkeys could be infected when injected percutaneously with the culture supernatant of transfected cells. Moreover, the intrahepatic injection of a full-length Sar55 clone transcribed in vitro into rhesus monkey and chimpanzees demonstrated that these constructs were also infectious in vivo^56,57^. The transfection of PLC/PRF/5, Huh7, Caco-2, and HepG2/C3A cells with viral RNA from the Sar55 strain resulted in its limited replication as measured by qPCR. However, no cell to cell spread occurred in these cells. The use of a Huh7-adapted cell clone (Huh7/S10-3) for Sar55 was also beneficial^58^. Meanwhile, infectious HEV cDNA clones are widespread. By using reporter genes such as GFP or luciferase, another readout concerning HEV subcellular localization or replication became possible^56,59,60^.

A more common method for propagating HEV in cultured cells is to inoculate suitable cells with viruses isolated from feces of acutely infected patients. Tanaka et al. used a genotype 3 patient isolate (JE03-1760F) with a genomic titer of 6.4 × 10^4^ and 8.6 × 10^5^ genomes/ml (ge/ml), respectively, to infect 21 cell lines. Among these cell lines, only A549 and PLC/PRF-5 cells supported viral propagation efficiently, peaking between 50 and 60 days postinoculation and reaching titers of approximately 10^6^ and 10^7^ ge/ml. Moreover, they showed that A549 and PLC/PRF-5 cells inoculated with a genotype 4 isolate (HE-JF5/15F) reached titers of approximately 10^8^ ge/ml^61^. Insertions in the hypervariable regions appear to occur more frequently than initially expected, some of which result in a growth advantage that makes them suitable for cell culture. The genotype 3 strain HE-JA04-1911, an infectious cDNA clone of a genotype 3 isolate (Kernow-C1 p6) passaged in HepG2/C3A cells that contained a 174 bp nucleotide insertion of human S17 ribosomal protein in the hypervariable region, replicated efficiently in cell culture and resulted in 75% positive cells. The same group described an S17 ribosomal protein insert (117 bp) that was also in the hypervariable region for the 2-hydroxybiphenyl-binding protein (HBPR), and Inoue et al.^62^ had previously identified a 39 nt insert in the HE-JA04-1911 strain^60,63,64^. A genotype 3c isolate (47832c) showed titers of up to 10^6^ ge/ml in A549 cells and 10^7^ ge/ml if the subclonal cell line A549/D3 was used^65^. However, an insertion in the hypervariable region of this clone was described recently^66^. It seems that these insertions occurred in the patient and were selected for during passaging due to a growth advantage^67^. It cannot be excluded that these mutations affect HEV biology. Thus, human embryonic or induced pluripotent stem cell-derived hepatocyte-like cells were used as cell culture models to study the HEV life cycle in vitro. These cell culture systems appear to allow the infection by non-adapted HEV isolates of genotypes 1–4 (ref. ^68^), but additional experiments are required to investigate the potential of these systems.

Taken together, all of the established cell culture systems currently available are inefficient. The best cells identified thus far for propagating HEV include A549, PLC/PRF-5, HepG2/C3A, and Huh7 cells or derived subclones. Moreover, the genotype 3-related strains are mostly reported to deliver high titers of infected or transfected cells.

### Animal models and reservoirs

Cynomolgus monkeys and chimpanzees were the first animals used for investigating HEV. Bayalan et al. transmitted enteric non-A and non-B hepatitis to cynomolgus monkeys (*Macaca fascicularis*) by intravenous injection of stool extracts from his self-experiment. Typically, the monkeys showed viral particles in the stool samples, elevated alanine aminotransferase levels and liver pathologies typical of acute hepatitis^12^. The infection of eight primate species (chimpanzees, four species of Old World monkeys, and three species of New World monkeys) resulted in seroconversion in all species except tamarins, while old world monkeys showed higher titers than new world monkeys. Investigations of wild monkeys and monkeys from breeding facilities showed already had a high seroprevalence for HEV infections^69^. The first successful vaccination against HEV was also performed in rhesus monkeys using truncated capsid protein for the prime/boost inoculation and previous infection with HEV^70,71^. The first animal HEV strain discovered was swine HEV in 1997 in the USA. It was reported that nearly all investigated pigs in the Midwestern USA were seropositive for anti-HEV IgG 3 months after birth, suggesting that swine HEV is widespread^48^. Domestic pigs and wild boars are the major reservoirs for zoonotic HEV genotypes 3 and 4. A review from Salines et al. in 2017 counted 86 studies (from 43 different countries) addressing the prevalence of HEV in farmed pigs. The studies documented a prevalence of viral RNA in the feces of domestic pigs between 10% and 100% and a seroprevalance between 30% and 98%^72^. Pigs infected with human-derived genotypes 3 and 4 were protected if already infected with genotype 3 swine HEV, showing a cross species protection^73^. No symptoms and only slight pathological abnormalities are detectable in pigs, but they are the most common source of HEV infections in industrialized countries.

In addition to monkeys and pigs, several other susceptible animals have been characterized. Rabbits were identified to host genotype 3 viruses and have shown their value as laboratory animals in a vaccine study^74,75^. A recent review carefully describes current small animal models for HEV^76^.

Bats found in Africa, Central America, and Europe were described as a host for HEV. An investigation of over 90,000 patient serum samples showed no bat HEV in the blood, and no recent articles have described an instance of transmission to humans.

A broad seroprevalence of rats against HEV has been reported for the USA, India, and Japan^77^. Evidence for rats carrying zoonotic genotype 3 and 4 strains was first described in the USA. A new rat-specific HEV strain was discovered recently in Germany and the USA that was distinct from all known genotypes and was therefore classified as orthohepevirus C1^78^. One study successfully infected athymic nude rats with the rat HEV strain LA-B350 to study the innate immune response, but these rats lack functional thymus and t-cells and are therefore limited as animal models^8^.

Primary hepatocytes from the tree shrew *Tupaia belangari* are susceptible to infection by a variety of human viruses, such as hepatitis B and C viruses. Experimental infection of tree shrews with swine HEV (genotype 4) by intravenous application with 1 × 10^5^ copies/mL showed that Tupaias are also prone to HEV infection^79,80^.

A recent report from a study in Spain detected HEV RNA that was closely related to genotype 3 in horses, donkeys, and mules, with respective prevalence of 0.4%, 1.2%, and 3.6%. Due to the low prevalence observed, García-Bocangera et al.^81^ concluded that equines are accidental hosts rather than a true reservoir.

Nonetheless, the described examples demonstrate that HEV is widespread among domestic and wild animals.

## VLPs

Since the production of high titer viral stocks using cell culture systems is difficult, many HEV entry/infection experiments have been performed using recombinant VLPs^82^. The expression of full-length capsid protein should result in a 71 kDa protein, but initial experiments producing the protein in insect cells showed that in addition to a 72 kDa protein, 58 and 50 kDa products could also be observed. The full-length protein is characterized by having poor solubility, but the N-terminally truncated 58 kDa (aa 112–660) and 50 kDa (aa 112–608) cleavage-product proteins were soluble and formed VLPs. These forms lack the N-terminal 112 aa that facilitates genome binding^83–85^. As the truncated version (aa 112–660) easily forms capsids, characterization by cryo-EM revealed a T1 symmetry formed by 60 monomers forming 30 dimers with a diameter of 27 nm^82^. Detailed structural analysis by X-ray crystallography led to the subdivision of the core protein into three linear domains. The S-domain, ranging from aa 118 to 313, is responsible for self-assembly; the P1-domain (aa 314–453) stabilizes the interaction of trimers; and the P2-domain, also designated as the E2s domain (aa 454–606), forms dimeric spikes and is exposed at the surface of the capsids. However, there is a second, slightly different definition of these domains. Yamashita et al.^86^ also divided the C-terminal half into three domains, designating them S- (aa 129–319), M- (320–455), and P-domains (456–606) that varied slightly from the division proposed by Guu et al.^87^ Dimerization of the capsid monomer to homodimers is a prerequisite for self-assembly and depends on the E2 domain (aa 394–606), especially on the hydrophobic amino acids Ala597, Val598, Ala599, Leu601, and Ala602. HEV-VLPs always show a T = 1 symmetry with a buoyant density of 1.28 g/cm^3^, unlike infectious virions from stool samples, which exhibit a T = 3 symmetry, a buoyant density of 1.290 g/cm^3^, and a diameter of 35–40 nm^88–91^.

The core protein contains three potential N-glycosylation sites (Asn137, Asn310, and Asn562) and various O-glycosylation sites. In cells overexpressing ORF2, glycosylation of the core protein can be observed^92^. A large quantity of the core protein is secreted and subsequently sialylated, N- and O-glycosylated. While mutation of the first two Asn-residues aborts self-assembly of the capsid, mutation of Asn562 allows particle formation^93^. For infectious viruses, glycosylation does not typically occur and is not relevant for viral release or entry^94^. One study showed that in an artificial Semliki Forrest Virus expression system, glycosylated HEV-core was less stable than the unglycosylated form, arguing for the hypothesis that the capsid is indeed unglycosylated^95^. A recent publication announced the formation of three different ORF2 forms: ORF2i (infectious/ intracellular), ORF2g (glycosylated), and ORF2c (cleaved ORF2). Only ORF2i was detectable inside cells as well as in infectious viral particles outside cells. The ORF2g and ORF2c proteins are highly secreted and display the most abundant antigens in patient sera, but they are not part of the infectious virus^94^.

The capsid is the target structure for neutralizing antibodies^82^. The E2s domain (aa 455–602) was also postulated to contain neutralizing epitopes^88^. A crystal structure of HE-VLPs showed that the exposed spikes of the capsids are formed by aa 456–606 of the ORF2 protein. Dimerization of E2s is crucial for attachment to the host cell and for binding of neutralizing antibodies^86^. The E2 and E2s domains form homodimers and oligomers that display conformational epitopes recognized by neutralizing monoclonal antibodies such as 8C11 and 8H3 (ref. ^96^). Detailed analysis revealed that aa 459–606 carry neutralizing epitopes in which six conformational antigenic sites could be identified^97^.

## Vaccines

The immunizing effect of the core protein was elucidated very early, and different strategies for developing an HEV vaccine have been studied. Monkeys (*Macaca fascicularis*) immunized with a 56 kDa capsid protein (Sar55) produced in insect cells that forms VLPs with T = 1 symmetry were protected from infection, showing no sign of hepatitis^71,98^. Rhesus monkeys vaccinated with 1 or 10 µg doses of the 56 kDa capsid protein were 100% protected from hepatitis and 63% protected from infection after the prime/boost application when challenged with an infectious dose of (10^4^) (ref. ^99^). A crystal structure of the E2s domain complexed with the neutralizing antibody 8C11 showed that Asp 496–Thr 499, Val510–Leu514, and Asn573–Arg578 are major interaction sites, with Arg512 being the most crucial one for neutralization^100^. In a subsequent phase 2, randomized, double-blind, placebo-controlled trial from Glaxo Smith Kline in Nepal with 2000 participants, the vaccine again based on the 56 kDa capsid protein protected effectively from HEV^101^. However, further development was terminated. A second approach is based on a shorter version of rHEV. This protein is termed p239 (aa 368–606) and displays a particulate structure^102,103^. The p239 protein was recombinantly produced in bacteria and purified under denaturing conditions, enabling the efficient removal of LPS. Importantly, the neutralizing epitopes were maintained after refolding^103^. By testing p239 as a vaccine candidate in Rhesus monkeys with a prime/boost approach of 5, 10, or 20 µg, the monkeys were protected after being challenged with HEV genotypes 1 or 4. The vaccine candidate was 100% effective at protecting monkeys from hepatitis and infection when a viral dose of 10^4^ genome equivalences was administered. By using a virus dose of 10^7^ ge, it was still 100% effective against hepatitis and 75% against infection^102^. A randomized, controlled phase II clinical trial with 457 adults showed a promising 100% seroconversion in the vaccinated individuals^104^. After establishing a robust scale up in production and lot increased consistency, a randomized, double-blind, placebo-controlled, single center phase III clinical trial was performed from August 2007 to June 2009 in China. Half of 112,604 individuals received the p239 vaccine based on genotype 1 and the placebo group received a hepatitis B virus vaccine by intramuscular injection of three doses (at 0, 1, and 6 months). The data showed that the vaccine was immunogenic, 100% efficacious, and well tolerated^105^. Based on this trial, p239 was licensed as Hecolin in China in 2012. However, several factors circumvented its approval in other countries or use by the WHO. The trial lacked children below 16 years of age and adults above 65 years of age as well as risk groups such as pregnant women and immunosuppressed patients. Moreover, protection against other genotypes (1/2/3) must be proven and the immunization schedule of three doses at 0, 1, and 6 months is not appropriate to counteract epidemics. A 4.5 year follow up study of the phase III trial and a phase IV trial with seniors over 65 years old helped the data to be interpreted a little better^106^.

The third candidate tested in clinical trial is also a bacterially produced self-assembling core protein (consisting of amino acids 439–617) called p179 produced by the Chanchun Institute of Biological Products Co. Ltd. First, animal experiments were performed by vaccinating rabbits to confirm its protective potential. Rabbits vaccinated with 10 mg of p179 produced anti-HEV with titers of 10^3^–10^4^ ge and were protected from hepatitis, but two out of the five rabbits exhibited viral shedding. In 2017, a phase I trial with p179 as vaccine was initiated with 120 participants to study its adverse effects and efficacy. The vaccine was well tolerated and appeared to be safe, and a phase II trial is ongoing^107^.

There are still a variety of open questions preventing the marked authorization of HEV vaccines outside of China. One interesting point to be resolved is why the Hecolin vaccine is so potent, since infectious viruses in the bloodstream are quasi-enveloped particles in which the antigenic core region is shielded from the humoral immune system.

## Conclusions

The understanding of the hepatitis E virus has changed over the last 15 years. For many years, HEV was considered to be a waterborne pathogen that causes hepatitis in resource-poor settings.

More recent studies clearly show that HEV is a relevant problem, even for developed countries, where it can be acquired zoonotically. In light of these findings, and considering that many HEV infections are asymptomatic, HEV is the focus of increasing attention with respect to the safety of blood and blood products. The portion of HEV RNA-positive blood products ranges from 1/726 for the Netherlands to 1/7431 for Australia^108,109^. In southeast England, screening of 225,000 blood donors for HEV RNA revealed that 79 donors were positive for HEV3. In 62 cases, the contaminated blood products were used before the HEV contamination was detected. Forty-three recipients were investigated, and HEV RNA was detected in 18 recipients (42%)^110^. Several cases of transfusion-transmitted infection were reported in France, Germany, Spain, and the United Kingdom (UK). A case–control study showed that markers of an acute HEV infection were detected more frequently in patients having received blood products (13/145) compared to the control group (2/250)^111^.

HEV has been transmitted through the application of red blood cells, platelet preparations, pooled granulocytes, and fresh frozen plasma. In Europe, Ireland, the UK, and the Netherlands have already implemented mandatory testing of blood donors, while screening of blood donations is under consideration in Germany^112,113^.

HEV is an underinvestigated virus for a variety of reasons. Thus, many aspects of the viral life cycle are not well characterized. One reason for this may be the lack of robust infection models, but significant progress has been made in this regard^10,11,105^. However, many open questions remain, with major unresolved issues including the need for a detailed characterization of the quasi-enveloped particle release pathway, the entry process involving a receptor (complex) that has yet to be identified and the processing of the viral polyprotein encoded by ORF1.

The lack of a licensed vaccine in most parts of the world and specific antiviral approaches affecting HEV replication to control of spread of HEV infections should be resolved in if research on HEV is intensified in the next years.
